# Methodologies for energy evaluation of pig and poultry feeds: A review

**DOI:** 10.1016/j.aninu.2021.06.015

**Published:** 2021-10-09

**Authors:** Jean Noblet, Shu-Biao Wu, Mingan Choct

**Affiliations:** a INRAE, UMR 1348 PEGASE, 35590 St-Gilles, France; b School of Environmental and Rural Science, University of New England, Armidale, NSW 2351, Australia

**Keywords:** Pig, Poultry, Feed, Energy value, Methodology

## Abstract

The cost of feed represents an important part of the total cost in swine and poultry production (>60%) with energy accounting for at least 70% of feed cost. The energy value of ingredients or compound feeds can be estimated as digestible (DE), metabolisable (ME) and net energy (NE) in pigs and ME and NE in poultry. The current paper reviews the different methods for evaluating DE, ME and NE of feeds for monogastric animals and their difficulties and limits, with a focus on NE. In pigs and poultry, energy digestibility depends on the chemical characteristics of the feed, but also on technology (pelleting, for instance) and animal factors such as their health and body weight. The ME value includes the energy losses in urine that are directly dependent on the proportion of dietary N excreted in urine resulting in the concept of ME adjusted for a zero N balance (MEn) in poultry. For poultry, the concept of true ME (TME, TMEn), which excludes the endogenous fecal and urinary energy losses from the excreta energy, was also developed. The measurement of dietary NE is more complex, and NE values of a given feed depend on the animal and environmental factors and also measurement and calculation methods. The combination of NE values of diets obtained under standardised conditions allows calculating NE prediction equations that are applicable to both ingredients and compound feeds. The abundance of energy concepts, especially for poultry, and the numerous feed and animal factors of variation related to energy digestibility or ME utilisation for NE suggest that attention must be paid to the experimental conditions for evaluating DE, ME or NE content. This also suggests the necessity of standardisations, one of them being, as implemented in pigs, an adjustment of ME values in poultry for an N retention representative of modern production conditions (MEs). In conclusion, this review illustrates that, in addition to numerous technical difficulties for evaluating energy in pigs and poultry, the absolute energy values depend on feed and animal factors, the environment, and the methods and concepts. Finally, as implemented in pigs, the use of NE values should be the objective of a more reliable energy system for poultry feeds.

## 1 Introduction

The cost of feed represents an important part of the total cost in swine and poultry production (>60%) and, in the feed, energy is the most expensive component accounting for 70% of feed cost (Noblet and van Milgen, 2004; Pirgozliev and Rose, 1999). This economic importance and effects of energy on animal performance have led to the development of different systems to express the energy value of feeds and the energy requirements of animals. In addition, the conflict for farmland use and feed crop production among different animal industry sectors, as well as for the competition among biogas, biofuels, and human foods, is becoming fierce in some parts of the world. This requires the definition of energy values of feeds and energy requirements of animals to provide effective facilitation for improved sustainability. However, the acquisition of energy values of feeds and determination of energy requirements of animals must be based on sound, reliable and accurate methods.

Not all gross energy (GE) that is consumed will be retained by the animal; there are losses in the faeces and urine, and as gases and heat. Based on these losses in the process of energy utilisation, different energy values and energy systems have been defined: digestible energy (DE) is the difference between GE intake and energy losses in the faeces; metabolizable energy (ME) is the difference between DE intake and energy losses in urine and gases from digestive fermentation, and net energy (NE) is the difference between ME intake and heat increment (HI). Depending on the collected energy-containing components (faeces and urine), either the digestible energy (DE) or ME can be determined for pigs, whereas, in poultry, the faeces and urine are excreted together, and hence the ME is commonly determined. In addition, in the collected energy components, a fraction originates from endogenous secretions and the rest from the consumed feed. Subtracting these endogenous losses from the total losses allows the calculation of true DE (TDE) or true ME (TME). These latter concepts were developed for poultry (NRC, 1994; Sibbald, 1982; Wu et al., 2020), but have been progressively abandoned due to the difficulty in estimating the contribution of endogenous losses to the excreted energy for fed animals. Most energy values used nowadays are then apparent DE (ADE) or apparent ME (AME) and, in practice, when referring to DE or ME values, it means ADE or AME values, respectively.

The main purpose of this review is to describe the main methods for characterisation of feeds for their energy values in pigs and poultry. This review should be complemented by the reviews by Zaefarian et al. (2021) on ME evaluation in poultry, and by Pirgozliev and Rose (1999) and Musigwa et al. (2021) on NE in poultry, and by Noblet and van Milgen (2004, 2013) and of Kong and Adeola (2014) on DE, ME and NE evaluation in pigs. The present review will then focus on NE but, as NE value is directly dependent on DE or ME content, the accuracy and reliability of NE values are dependent on the methods implemented at all steps of energy evaluation.

## 2 Gross energy of feeds

The heat of combustion, or GE, is the most basic form in which energy can be expressed and is a property of the feed itself. The GE content of a feedstuff can be evaluated by burning a sample of <1 g in a bomb calorimeter and measuring the heat released. The measurement is usually done on a compacted sample (i.e., pellet); alternatives are available for liquid products (liquid fat, for instance). The accuracy (+/− 10 kcal or ± 40 kJ per kg) and repeatability of GE measurement are high. The GE content of raw materials varies greatly and ranges from about 15 kJ/g dry matter (DM) for sugar cane molasses to 39 kJ/g DM for oils and fats (Sauvant et al., 2004). The difference in GE content between feeds is due to differences in chemical composition and chemical bonds. In the absence of a bomb calorimeter, the GE values of feeds may be estimated from the chemical composition using prediction equations (Sauvant et al., 2004). For instance, the GE (kJ) of feeds can be predicted by an equation that includes all energy-yielding nutrients (g). The following equation was obtained from measurements of 191 complete diets by Noblet et al. (2004):

GE = 23.0 × CP + 38.9 × EE + 17.4 × Starch + 16.5 × Sugars + 18.8 × NDF + 17.7 × Residue ,

where “Residue” is the difference between total organic matter (OM; i.e., DM minus ash) and the other identified fractions in the equation. Although this equation is empirical, it reflects the energy value of individual nutrients very well. A comparable but simpler equation calculated from more than 600 results allows to calculate the GE in pig faeces when no bomb calorimeter is available:

GE (MJ/kg DM) = 18.73 – 0.192 × Ash + 0.223 × EE + 0.065 × CP ,

with chemical indicators as % of DM (Noblet and Jaguelin-Peyraud, unpublished data). This equation can also be applied to poultry excreta.

In conclusion, the GE measurement of feeds (complete diets or ingredients) and excreta is rather easy and quite accurate. However, as for any measurement, the bomb calorimeter must be correctly calibrated – benzoic acid is routinely used for calibration - and the small, pelleted sample that is burnt must be representative of the total feed batch. According to the extreme energy contents of ash (0 kJ/g) and EE (#40 kJ/g), attention should be paid to get a sample whose ash and EE contents are as close as possible to the ash and EE contents of the total batch.

## 3 Measurement of DE and ME values of complete feeds for pigs

### 3.1 Digestible energy

The DE content of a feed sample corresponds to its GE content minus energy losses after digestion and is obtained as GE of feed consumed minus that in the faeces. Even though they are related to digestion, the energy of gases and heat originating from hindgut fermentation are not considered in the calculation of DE. The ratio between DE and GE corresponds to the digestibility coefficient of energy (DCe, %) and is equal to [100 × (GE intake – GE faeces)/GE intake].

The basic methodology for measuring DE content of feeds in pigs consists of keeping the pigs individually in digestibility pens and adapting them to the pen and to the feed for at least 5 d and up to 14 d—a longer adaptation period is recommended for high fibre feeds and heavy or adult animals—before a total collection of the faeces over 2 to 10 d, with 5 d being sufficient in most situations (Liu et al., 2020). However, the longer the collection period, the more accurate is the DE estimate. The basic measurements consist of: 1) quantifying the feed DM intake (as proposed feed multiplied by percentage DM at weighing of feed minus dry feed refusals, wastage, etc.) and fecal DM excretion (as freshly homogenised faeces multiplied by their DM content at weighing of faeces), and 2) measuring the GE and DM of representative samples of feed and faeces and expressing their GE contents on a DM basis. In addition, since the only stable and easily controllable indicator of feed intake is the feed DM intake, it is absolutely necessary to establish the ingredient composition of the diet on a DM basis and then to measure the DM content of all ingredients at the time of their mixing for getting the final feed that will be offered to the pigs. That is particularly important for evaluating the DE and ME content of ingredients (discussed more later). In such digestibility studies, the animals are usually meal-fed at feeding levels equivalent to about 90% of their ad libitum intake in order to harmonise the feeding levels between animals and treatments, to avoid refusals and wastage and get a regular transit of digesta and fecal excretion.

Energy digestibility in pigs is affected by diet composition, diet presentation (higher for pellet than for mash, particle size, etc.), BW of pigs with higher values in heavier and/or older animals, feeding level (Le Gall et al., 2009; Le Goff and Noblet, 2001; Noblet and van Milgen, 2013) and pig genetic characteristics (Noblet et al., 2013). This means that different DE values should be applied according to the technology of preparation of feeds and, more importantly, the stage of production. Practically, at least 2 sets of energy values are used in feeding tables and feed formulation, i.e., that applicable to: 1) piglets and growing-finishing pigs, and 2) adult sows (Noblet and van Milgen, 2004). It is then important to harmonise and control these animal and technological factors within a trial or take into account their effects when comparing trials or data obtained under variable experimental conditions. For end-users, attention should also be paid to the experimental conditions when literature DE values are implemented in formulation matrices. Similarly, according to the regular increase of DCe with BW increase in growing pigs (Table 1), DE values in growing pigs should be preferably obtained in about 60 kg BW pigs in order to be applicable to the total growing-finishing period (Noblet and van Milgen, 2004, 2013).

**Table 1:** Effect of pig body weight on energy digestibility.^1^

Stage	BW, kg	DM intake, g/d	Energy digestibility, %
1	38	1,250	82.6
2	49	1,680	83.0
3	61	1,940	83.6
4	72	2,015	84.2
5	80	2,060	84.8
6	90	2,120	85.3
Total growth	35 to 95	1,845	83.6

1 Mean values obtained on 4 diets based on wheat and soybean meal and variable proportions of wheat bran, rapeseed oil and animal fat; measurements were carried out continuously (5 successive 8–10 d periods) on the same pigs from 35 to 95 kg (5 pigs per diet); the effect of BW (or period) on energy digestibility was significant (*P* < 0.01); the interaction between pig stage and diet composition (i.e., fibre level) was also significant (*P* < 0.01) (J. Noblet, unpublished data).

In the formulation of pig feeds, the vitamin and mineral premix (MV) is considered as providing no energy and is therefore equivalent to a diluent as far as energy is concerned. However, the ash content of the feed originating from energy-yielding ingredients and/or MV have an effect on DE content or DCe that is greater than the simple dilution effect (Noblet and Perez, 1993). The impact of minerals such as dicalcium phosphate and calcium carbonate on DCe or DE value of the energy-yielding ingredients is illustrated in Table 2. In other words, in digestibility trials, it is important to keep constant the level and composition of MV across all diets with levels of inclusion relevant to practice: the negative effect of ash is then affected to all energy-yielding ingredients included in the diet. This may also explain some of the effects of phytase addition and subsequent lower dietary P level on additional energy and amino acid digestibility (Adeola and Cowieson, 2011).

**Table 2:** Effect of ash addition on energy digestibility in 60 kg growing pigs (J. Noblet, unpublished data).

Diet	1	2	3
Ingredients			
Basal diet^1^, %	99.0	96.0	93.0
Dicalcium phosphate, %	0.5	2.0	3.5
Calcium carbonate, %	0.5	2.0	3.5
Feed intake, g DM/d	1,886	1,893	1,913
Energy digestibility^2^, %	85.5	83.4	82.4

1 Basal diet contained corn (26.4%), wheat (26.4%), barley (26.4%), soybean meal (20.0%), salt (0.4%) and oligo-elements and vitamins (0.4%).

2 Energy digestibility differed (*P* < 0.01) between the 3 diets.

The containment of pigs in metabolic cages, even with free movement, may be problematic from a welfare point of view. Therefore, it may be necessary to estimate digestibility coefficients of energy and nutrients in animals kept in pens (and in groups). It is also laborious to precisely measure feed intake and faecal excretion over a rather long period of time in cage-housed pigs. Using an indigestible marker included in the feed with a grab sampling of excreta per pig allows implementing digestibility studies under conventional housing conditions (in groups, free-moving, ad libitum feeding, etc.) on the basis that the totality of the marker included in the feed is excreted in the faeces. This means that the quantity of DM (kg) excreted per kg of DM feed intake is equal to the marker content in the feed DM (Mf) divided by the marker content in the excreta DM (Me). The DE content of the feed DM then equals its GE (GEf) minus the corresponding energy output in the faeces (Mf/Me) multiplied by the GE content of the excreta DM (GEe). Therefore, DE = GEf – (Mf/Me) × GEe and DCe (%) = 100 × [1 – (Mf/Me) × (GEe/GEf)]. The advantages and disadvantages of this attractive method and the potential markers have been discussed in several papers (Kong and Adeola, 2014). In brief, the marker must be totally undigested, uniformly distributed in the feed and the faeces and, most importantly, it can be easily and accurately measured in both feed and the faeces at relatively low concentrations (<0.5%). The accuracy and repeatability of this methodology may then be variable with the quantity and representability of the faecal sample and the accuracy of the marker analysis in the feed and the excreta. Overall, the accuracy of DE determinations is lower for the marker method than for the total collection. The comparison of DE values obtained by the total collection and marker method does not indicate any systematic difference between the methods and between the markers (Huang et al., 2018). It should also be noted that the marker method can be used in pigs kept in digestibility cages, avoiding tedious and laborious collections of total excreta over a few days. Apart from evaluating the digestibility of energy and nutrients, coloured markers can also be used for estimating the start and the end of the excreta collection (Li et al., 2016). This coloured marker technique is not frequently used for studies requiring longer collection periods and that needing moderate fasting periods before and after the collection.

### 3.2 Metabolizable energy

The ME content of a feed is equivalent to the difference between the DE content and energy losses in urine and as gases (mainly methane in pigs). Urine is collected in digestibility crates after separation from the faeces and, much less frequently, in adult female pigs (pregnant or lactating) equipped with bladder catheters. Urine must be collected over a minimum of 3 d. As for faeces, the energy content of urine can be measured using a bomb calorimeter after freeze-drying an aliquot. However, this operation is rather laborious and consumes a lot of time with an end result that is at best moderately accurate. Therefore, equations for predicting urinary energy (MJ per kg feed DM) from urinary N (Nuri; grams per kilogram of DM feed intake) have been proposed. From a compilation of 610 measurements, the following equation was established in growing pigs: Urinary energy = 0.19 + 0.031 Nuri. A comparable equation has been proposed for adult pigs (Le Goff and Noblet, 2001; Noblet et al., 2004).

The excretion of N in the urine depends on the difference between digestible N and retained N, which, in turn, depends on the quantity of protein in the feed and the capacity of the pig to retain energy or export energy (milk) as protein. The urinary energy can therefore vary according to the physiological stage of the pig and the diet characteristics. In practice, the application of a single ME value to a compound feed or a raw material is convenient. Hence it is suggested that urinary energy losses be standardised, which, in turn, be used to standardise ME values using a urinary N loss calculated as a constant proportion, i.e., 50%, of digestible N or 40% of total N (Noblet et al., 2004). This then implies that most ME values of feeds published in the literature and feeding tables may be incorrect and should therefore be considered with caution. This is particularly important in high protein feeds for which N excretion is in excess of what will occur with balanced and low protein diets with subsequent underestimated ME contents. This point will be discussed in more details later in the Ingredients Section.

The measurement of methane production necessitates the pig to be housed in a respiration chamber. In addition, the energy loss as methane is small in piglets and growing pigs (<0.5% of DE) (Li et al., 2018a; Noblet et al., 1994b) and is therefore neglected in most situations. However, in adult pigs where hindgut fermentation is higher, especially with high fibre diets (Ramonet et al., 2000), than in young pigs, methane production is 4 to 5 times greater than in growing pigs (up to 2% of DE) and thus deserves consideration in ME evaluations. In the absence of respiration chambers, indirect methods based on the quantity of digestible dietary fibre allow estimating energy losses as methane in pigs (Noblet et al., 2004).

## 4 Measurement of ME values of complete feeds for poultry

Unlike pigs, the faeces and urine are excreted together in poultry. Therefore, it is convenient to measure ME, rather than DE. ME is obtained as GE intake (GEf) minus GE of excreta, which is the pool of faeces and urine (GEfu). Gaseous energy loss is very low in poultry and is usually neglected. The concept of true ME (TME) has been developed in poultry considering that a fraction of excreta energy losses is of an endogenous origin (GEend) at both the intestinal (including bile, enzymes, mucosal cells) and urinary (including protein metabolism residues) levels. These losses are not related to feed characteristics. The endogenous fraction is then subtracted from GEfu to derive TME, i.e., TME = GEf – (GEfu – GEend). This TME concept was intensively studied and developed in North-America (Sibbald, 1982), but it has been progressively replaced by the apparent ME (AME) system with no consideration of endogenous energy losses (Bourdillon et al., 1990b). Another point for poultry is that TME and AME values are often corrected for a zero N balance of the birds (TMEn or AMEn, respectively) in order to standardise the ME values between birds retaining variable proportions of their N intake and adjust the ME values to the level of adult cockerels which do not retain any N (Bourdillon et al., 1990a). A coefficient of 8.22 kcal/g N gain (or 34.4 kJ) corresponding to the energy content of uric acid per g of N is used (Hill and Anderson, 1958). The N gain can be estimated from BW gain considering that BW gain contains 20% crude protein or 3.2% N (i.e., 20/6.25) (Carré et al., 2014).

Nowadays, most literature ME values (Wu et al., 2020) and recent feeding tables (CVB, 2018; Rostagno et al., 2017) for poultry are based on the AMEn values from measurements conducted mostly in broilers, although data obtained in adult cockerels still remain in some feeding tables. However, the metabolisability of energy (AMEn/GE) differs owing to production stages, ages and species in poultry (Table 3) (Cozannet et al., 2010; Stefanello et al., 2016) with potential confusions and misuse of literature values. In addition, the zero N balance correction looks meaningless if we consider that all productive birds (broilers, layers, and turkeys) retain up to 60% of their N intake (Barzegar et al., 2019b; Wu et al., 2019). As done in pigs (Noblet et al., 2004), it would then be logical to standardise the AME values (AMEs) for a positive N balance representing, for instance, 50% of the N intake: AMEs = AME + 8.22 × (0.50 × N intake - N gain) if AME in kilocalories and N in grams (34.4 instead of 8.22 if in kilojoules, Table 3) and 50% N retention (Cozannet et al., 2010).

**Table 3:** Effect of production stage and species on metabolisability of energy in poultry (from Cozannet et al., 2010).^1^

Item	Rooster	Broiler (3 weeks)	Laying hen	Turkey (10 weeks)
DM intake, g/d	65	77	87	349
AMEn, % GE	69.0^a^	65.3^c^	66.4^b^	64.3^c^
AMEs, % GE	72.6^a^	68.9^c^	69.9^b^	67.9^d^

1 Eleven diets based on wheat, corn, SBM and wheat DDGS; effect of stage/species: *P* < 0.001; AMEn and AMEs standardised for retained N equal to 0% and 50% of N intake, respectively.

Methods for evaluating ME in poultry have changed over time, both for animals used (adult rooster, growing broiler, and laying hen) and feeding techniques (ad libitum, restricted feeding, and force-feeding) in the studies. As for pigs, direct measurement of total feed intake and faecal output over a few days, as known as the total collection method, and spot sampling of the faeces containing an indigestible marker, known as the marker/indicator technique, are routinely used in different forms. The use of adult roosters was introduced in the 1970s to 1980s (Farrell, 1978; Sibbald, 1982) but it has now been almost totally phased out due to a number of concerns, including animal welfare issues and the disconnect between the value obtained with the practical feed formulation (Härtel, 1986). For the same reason, the egg industry in recent years has emphasised the importance of using birds of relevant age and physiology hens when determining the ME values for laying hens (Barzegar et al., 2019b). The force-feeding technique was used for TME measurements with a low quantity of feed (complete feed or ingredient) and a short duration of excreta collection (Sibbald, 1982), whereas most studies today are conducted in *ad libitum* fed birds. The duration of excreta collection is also rather variable (1 to 5 d) with 3 to 7 d of adaptation period as well as a fasting period ranging between 6 and 15 h before starting and completing the actual excreta collection; the fasting periods allow to start and end the collection period with a nearly empty digestive tract, minimising any bias from feed consumed immediately before or after the collection period (Wu et al., 2020). As for pigs, attention must be paid to the maintenance of constant level and composition of MV, the expression of diet ingredient composition as DM basis, the precise determination of DM intake and DM excretion, and laboratory analyses expressed on a DM basis. Finally, and same as pigs, the AME value of complete feeds varies with age of the birds (Stefanello et al., 2016) and their production stage (Table 3), the form of feed (pellet vs mash; Khalil et al., 2021; Pirgozliev et al., 2016) and the use of feed supplements, such as enzymes (Kiarie et al., 2017; Ravindran, 2013). All these factors contribute to the variation of the AME value and hence must be standardised within a trial (or a series of trials) so that energy values obtained under different experimental conditions can be compared.

In conclusion, the most common methodology presently used for ME evaluation in poultry includes growing broilers (15 to 30 d of age) fed ad libitum, adapted to the feed and the cage for at least 3 d and feed intake and excreta output measured for 2 to 5 d. The same conditions should prevail when inert markers are used to measure ME (Wu et al., 2020).

## 5 Prediction of DE and ME values of complete feeds for pigs and poultry

The DE and ME contents of diets can be obtained as the cumulative DE or ME contributions of the ingredients included in the complete feed. The energy values for the ingredients can either be obtained from feeding tables or by other techniques (see next section). This assumes there are no interactions between ingredients or among nutrients, a concept well-accepted for formulating pig and poultry diets. However, the actual ingredient composition of the feed is often unknown, and hence alternative solutions are used.

The first group of solutions is based on prediction equations of DE in pigs and ME in pigs or poultry from major chemicals present in complete diets (Carré et al., 2013; Le Goff and Noblet, 2001; Noblet and Perez, 1993). As an example, the following equation was developed by Le Goff and Noblet (2001) from a compilation of measurements conducted on 77 diets fed as mash to 60-kg pigs:

DE (MJ/kg DM) = 17.69 + 0.146 EE + 0.071 CP – 0.132 NDF – 0.341 Ash (RSD = 0.31 MJ) ,

with chemical contents expressed as percentages of DM, and EE and CP are ether extract and crude protein, respectively. The coefficients of that equation illustrate the predominant roles of fat (positive) and dietary fibre and ash (negative) on DE and ME predictions. As indicated above, the coefficient for ash is much higher than a simple energy diluting effect. The validity and the feasibility of such equations are dependent on the size and variability of the database used for their calculations and the accuracy and the statistical significance of each coefficient. Unfortunately, some publications propose equations with redundant or correlated predictors (GE and fat or NDF and ADF, for instance) and no indication of the statistical significance of the coefficients of the equations. More importantly, as indicated above for pigs and poultry, the DE and ME values of a compound feed vary with the animal BW, its production stage and the technology of feed preparation. Such equations should then be established from measurements conducted according to a standardised methodology and, in theory, they should not be applied afterwards to conditions that differ from the original conditions and premises on which the measurements were taken and calculations based. Such an important prerequisite is often forgotten. Similarly, these equations have been obtained with complete feeds that often contain higher levels of ash than single ingredients and the mean chemical, and physical characteristics of fibre in the database values may differ markedly from those of a given ingredient. Therefore, equations obtained on complete feeds, especially those including an intercept (as above) should never be applied to single ingredients (Cemin et al., 2021). More generic equations based on the average DE or ME contributions of each energy-yielding nutrient may then be applied to both complete feeds and ingredients; the energy contribution of ash is then considered as nil and/or the negative effect of ash on dietary DE or ME values is considered for all nutrients. Again, the coefficients obtained will represent the average contributions of nutrients across a large number of ingredients. On the same set of 77 diets mentioned above, Le Goff and Noblet (2001) proposed the following equations for pigs:

DE (MJ/kg DM) = 0.225 CP + 0.317 EE + 0.172 Starch + 0.032 NDF + 0.163 Residue (RSD = 0.35 MJ) ,

ME (MJ/kg DM) = 0.201 CP + 0.318 EE + 0.171 Starch + 0.026 NDF + 0.165 Residue (RSD = 0.35 MJ) ,

with “Residue” as the difference between OM content and the other nutrients considered in the equation (% of DM). Comparable equations are obtained from a recompilation of the AME values of 30 diets obtained in 4-wk broilers by Carré et al. (2013):

AME (MJ/kg DM) = 0.227 CP + 0.325 EE + 0.160 Starch – 0.065 NDF + 0.070 Residue (RSD = 0.26 MJ) ,

AMEn (MJ/kg DM) = 0.207 CP + 0.322 EE +0.157 Starch – 0.063 NDF + 0.068 Residue (RSD = 0.25 MJ) .

The comparison of these 2 sets of equations in pigs and poultry indicate comparable DE or ME contributions of the 3 major energy-yielding nutrients (fat, starch and crude protein), no energy contribution of dietary fibre in poultry and, to a smaller extent, in pigs, a much higher contribution of the so-called Residue (i.e., sugars, soluble dietary fibre, etc.) in pigs than in poultry and a logical decrease of the energy contribution of crude protein when moving from DE to ME in pigs or AME to AMEn in poultry.

The second group of methods is based on in vitro techniques which consist of simulating in “test tubes” the successive steps of in vivo digestion. This is used for different nutrients and, in the case of energy, the measurement is usually based on in vitro digestibility of OM (dOMv) and the relationships between energy digestibility and dOMv. Such methods have been widely used for ruminants but much less for poultry (Zaefarian et al., 2021; Zhao et al., 2014) and pigs (Boisen and Fernández, 1997; Noblet and Jaguelin-Peyraud, 2007) to assay complete feeds. The method of Noblet and Jaguelin-Peyraud (2007), adapted from the original method of Boisen and Fernández (1997), was evaluated on different sets of feeds (complete feeds vs ingredients; mash vs pellet; growing pigs vs adult pigs) for predicting dOMv and DE values. The predictions of dOMv and DE values were satisfactory in the case of complete feeds (*n* = 79) as well as ingredients (*n* = 66) when fed to young growing pigs as mash. However, it was unable to produce a satisfactory prediction of DE from dOMv for processed feed (pellet) or in adult pigs. In other words, the effect of any technological (pellet vs mash) or animal (adult vs young) factor on in vivo digestion could not be measured using the dOMv method. This also means that in vitro digestion methods may not be able to quantify the effects of factors such as supplemental enzymes, while it is rather frequently used for a rapid evaluation of their effects, either in pigs or in poultry (Vangsøe et al., 2021). Finally, the in vitro prediction of energy values should be used with caution when it is applied to feeds where preparation (pelleting, enzymes, etc.) and animal target differ from the experimental conditions under which the prediction equation was established. In the case of poultry, some in vitro methods have been proposed, but their validation and comparing in vivo and predicted values remain unsatisfactory (Zaefarian et al., 2021).

The third method available to predict DE and ME values is based on the measurement of the near-infrared (NIR) spectra of feed; the principle of this method is more or less equivalent to the prediction from chemical composition. However, it is based on the amount of light absorbed by certain chemical bonds in the specific infrared wavelengths and is non-destructive to the sample. One major advantage of the NIR method is its speed, simplicity and low cost. However, the accuracy of the DE and ME predictions depends on reliable in vivo DE and ME values used for calibrating the NIR machine. Otherwise, it is “rubbish in rubbish out” as the NIR spectroscopy is a blackbox technology requiring precise calibration and regular validations. Attempts have been made to develop NIR spectroscopy predictions for complete feeds in pigs (Aufrère et al., 1996) and poultry (Losada et al., 2009). However, NIR spectroscopy calibrations are more successfully used for single ingredients (wheat and its by-products, for instance) (Li et al., 2016) than for complete feeds.

As for DE or ME prediction from chemical indicators, the NIR method is not able to take into account the effect of technological preparation (pelleting, for instance) or the presence of supplements in the feed (enzymes, etc.). Similarly, the prediction is valid only for the experimental conditions under which the in vivo measurements were taken. For instance, a prediction based on AMEn measured in adult cockerels is unable to predict the AMEs in broilers; likewise, that based on the DE values in growing pigs won't be able to predict DE in adult pigs accurately. In other words, an NIR prediction of DE or ME should be accompanied by the experimental conditions where in vivo data were collected for the calibration in the first place. This is particularly important for poultry with confusions related to adjustment for N balance, the age of the animals (adult vs broiler), and the ME types (AME vs TME). Unfortunately, these are frequently ignored in commercial application and research alike.

The fourth group of methods consists of using the NIR spectra of excreta for predicting the DE or AME values of diets. The excreta represent the end-product of the digestive process, and its composition is dependent on both the feed itself and the history of its transit through the animal digestive tract. The analysis of excreta can then be indicative of either the feed composition (for undigested or low digestibility components) or the extent of OM digestion by the animal. As indicated above, the NIR spectroscopy is able to produce a global view of the faecal or excreta chemical composition, which means that it can provide a good estimate of the extent of OM digestion for a given feed. This methodology has been implemented in most animal species, including poultry (Bastianelli et al., 2010) and pigs (Bastianelli et al., 2015; Schiborra et al., 2015). This technology has a particular interest in genetic studies concerning a high number of animals housed under conventional conditions, fed a single diet and evaluated for their genetic capacity for feed digestion. So far, it has been used in genetic studies in pigs (Déru et al., 2021; Nirea et al., 2018; Noblet et al., 2013) and poultry (Mignon-Grasteau et al., 2004) that were fed a single diet. A major advantage of this methodology is that a grab sample obtained in free-moving animals kept in pens is sufficient; that is particularly interesting in large size animals like pigs. As for NIR prediction from feed spectra, the calibration is based on in vivo measurements of DE or ME and excreta spectra with the same feed.

In conclusion, the prediction of DE and ME values of complete feeds for pigs and poultry is possible without any in vivo measurement on animals. However, the accuracy of predictions varies between the methods and is directly dependent on the potential of the methods to evaluate: 1) the level and the nature of dietary fibre acting as an energy diluent, 2) the level of ash, and 3) the level and, to a lesser extent, the composition of fat. In addition, these methods may have limits for their application, with some of them being not valid for ingredients.

## 6 Evaluation of DE and ME contents of ingredients in pigs and poultry

Some ingredients (e.g., cereals) can be fed alone to pigs or poultry, and the measurement of their DE or ME value is quite similar to what is described above for complete feeds. However, many ingredients can only be included in limited amounts in a diet to meet animal tolerance and/or practical and commercial relevance. For instance, depending on the type of diet, the fat inclusion level should be below 6% to 8%, the dietary fibre sources below 20% to 25% in growing pigs and 10% to 15% in broilers, and most protein sources below 25% to 30% in pigs and poultry. In these circumstances, the evaluation of DE or ME contents of the ingredients becomes indirect with concomitant measurements of DE or ME of one or several ingredients in complete feeds excluding the ingredient(s) in question as basal diets (B diet) or including the ingredient(s) in question as test diets (T diets) at practical levels. The general principle is that the GE, DE or ME of the complete feeds are associated with the inclusion levels of the ingredients and their respective GE, DE or ME values. Strategies can be applied to simplify the calculations with the simplest being the so-called difference or substitution method.

All methodological considerations developed for the DE or ME measurement of complete feeds (see above) should be applied to the measurements of diets for evaluating DE or ME of ingredients: minimal durations for adaptation and excreta collection, total collection or marker-based method to measure excreted energy in faeces or excreta, the precise ingredient composition of feed at its preparation (i.e., DM relative to DM), constancy of the MV mixture level in all diets, accurate measurement of DM in excreta (faeces in pigs, faeces + urine in poultry) relative to feed DM intake (i.e. digestibility or metabolisability of DM) and the accurate analyses of feeds (diets and ingredients) and excreta expressed on DM basis. The flaws in the experimental designs, the methodological approaches and, most importantly, the calculation methods have been discussed in several reviews (Kong and Adeola, 2014; Noblet and van Milgen, 2013; Wu et al., 2020). The following sections will describe the most appropriate and correct methods for a reliable estimate of the DE or the ME values of ingredients.

### 6.1 Difference or substitution method

In the simplest version of the difference method, a B diet meeting the animal's requirements and a T diet where a fraction of the B diet is replaced by the test ingredient are included in the assay. The GE, DE and ME values of diets are then measured. Several T diets can be prepared with one B diet within a trial in order to evaluate several ingredients simultaneously. It is then assumed that the difference in the measured GE, DE or ME contents between one T diet and the B diet is due only to the test ingredient inclusion (i.e., no interaction) or that the energy value of the B diet is identical in both B diet and T diet. It is also assumed that the fraction of minerals and vitamins (MV) in the diet does not provide energy. However, as indicated above, the DCe depends on the ash content in the diet with the ash effect being higher than a simple dilution effect. Therefore, it is important to have a constant MV fraction in the B and T diets. In its simplest version, only one B diet is prepared and a fraction of this B diet (excluding MV) is replaced by a test ingredient which may produce imbalanced, and in some cases, extreme diets. For instance, elevated levels of substitution of B diet with high protein ingredients will result in very high protein diets, leading to excessive protein catabolism, disturbance of feeding behaviour and potential digestive disorders. An alternative solution consists of preparing 2 B diets containing one cereal (corn, for instance) and one protein-rich ingredient (soybean-meal, for instance) but at different inclusion levels in order to obtain 2 B diets at a low and a high protein level in the range tolerable by the animal. The DE and ME values of corn and soybean meal can be obtained from the DE and ME values of the 2 B diets by solving a system of 2 equations with 2 unknowns. The T diets can then be prepared with one test ingredient and different combinations of the cereal and the high protein source used in the B diets in order to obtain acceptable levels of crude protein in the T diets. As for the simplest difference method (one B diet), the GE, DE or ME values of each test ingredient are calculated by subtracting the calculated GE, DE and ME contributions of the cereal and the protein source used in the B diets according to their inclusion levels (DM relative to DM).

In the simple difference method, the first estimate of GE or DE or ME (Eti_0_ for GE_0_, DE_0_ or ME_0_; per kg DM) of the test ingredient is calculated as:

(Eq. 1) Eti_0_ = {Etd – [Ebd/(1 – %MVbd)] × (1 – %ti – %MVtd)}/%ti

following the assumption that Etd = Eti_0_ × %ti + [Ebd/(1 - %MVbd)] × (1 - %ti - %MVtd) and where Etd and Ebd are the measured GE, DE or ME values of the T diet and the B diet (per kg DM), respectively, %ti is the percentage unit of the test ingredient in the T diet (DM/DM), and %MVbd and %MVtd are the percentage units of the minerals and vitamins mixture (DM/DM) in the B diet and T diet, respectively. This formula allows calculating the DE/GE (DCe, %), ME/DE (%) and ME/GE ratios for the test ingredient as:

(Eq. 2) DCe (%) = 100 × DE_0_/GE_0_ = 100 × {DEtd – [DEbd/(1 – %MVbd)]} × (1 – %ti – %MVtd)/{{GEtd – [GEbd/(1 – %ti – %MVbd)]} × (1 – %MVtd)} ,

(Eq. 3) ME/DE (%) = 100 × ME_0_/DE_0_ = 100 × {MEtd – [MEbd/(1 – %MVbd)]} × (1 – %ti – %MVtd)/{{DEtd – [DEbd/(1 – %ti – %MVbd)]} × (1 – %MVtd)} ,

(Eq. 4) ME/GE (%) = 100 × ME_0_/GE_0_ = 100 × {MEtd – [MEbd/(1 – %MVbd)]} × (1 – %ti – %MVtd)/{{GEtd – [GEbd/(1 – %ti – %MVbd)]} × (1 – %MVtd)} ,

The DE value of the test ingredient in pigs is then calculated as its GE as measured in the laboratory multiplied by calculated DCe (Eq. (2)) and ME value is obtained as DE multiplied by calculated ME/DE (Eq. (3)); for poultry, ME is obtained as measured GE multiplied by calculated ME/GE (Eq. (4)).

Other calculation formulas have been proposed in the literature. For instance, Adeola (2001) and Kong and Adeola (2014) proposed to calculate DCe of a test ingredient (DCe-ti) as:

(Eq. 5) DCe-ti = DCe-bd + (DCe-td – DCe-bd)/Pti ,

where DCe-bd and DCe-td are the digestibility coefficients (% or percentage units) of energy in the B diet, and the T diet, respectively, and Pti is the fraction of GE in the T diet provided by the test ingredient. The same formula is used for calculating ME/GE of poultry (and pig) diets by replacing DCe with ME/GE. The difficulty and potential source of errors in using that latter formula is the correct calculation of Pti that should take into account the DM contents of ingredients when preparing the basal and test diets and determining the GE value of each ingredient.

Unfortunately, as described in detail in the review of Wu et al. (2020) for poultry ME, this basic calculation method is not fully implemented with several simplifications and errors. First, the so-called “difference method” may be cited but without any detailed description or reference, and it is then impossible to get a critical appreciation of the result. Second, the proportions of ingredients used in the B diet are slightly different from that in the T diet, and/or the levels and composition of MV are changed; some minor ingredients such as free amino-acids may also be used differently in the B and T diets. Third, unintentional simplifications are used with no measurement of the DM content of the ingredients at diet preparation and then assuming that the DM content of all ingredients is identical. Fourth, the most frequent error in DE or ME evaluation of ingredients according to the so-called “difference method” consists of proposing DE or ME values of the test ingredient equal to DE_0_ or ME_0_ as calculated from Eq. (1) with no control of the corresponding GE_0_ of the test ingredient that can be calculated from GE values measured on the B and T diets used in Eq. (1). Unfortunately, GE of the test ingredient as measured on the test ingredient alone differs to variable extents from the calculated GE_0_ in connection with incorrect measurement of GE of B and/or T diets and/or incorrect sampling of B and T diets and/or insufficient mixing and homogeneity of B and T diets. As illustrated in Table 4 for vegetable fat and barley in poultry diets, any discrepancy between GE and GE_0_ is accompanied by a corresponding discrepancy between DE_0_ and DE (or ME_0_ and ME). This means that: 1) GE_0_ and GE of the test ingredient should be compared in order to detect potential methodological errors due to mixing and homogeneity of diets, to analytical procedures for GE and to calculation methods; and 2) assuming DE (or ME) equal to DE_0_ (or ME_0_) is erroneous. The calculation of DCe-ti or ME/GE-ti as indicated in Eqs. (Eq. 2), (Eq. 4)), even unable to produce correct GE values of feeds, attenuates the impact of such methodological flaws in the calculation of reliable DE or ME values (Table 4).

**Table 4:** Energy values of soybean oil and barley according to the difference method in broilers: impact of GE measurement errors on diets on calculated energy values of ingredients.^1^

Item	Basal diet	Test diet 1/Soybean oil	Test diet 2/Barley
Ingredients, % DM			
Corn	64.2	60.4	50.6
Soybean meal	31.4	29.6	24.8
Soybean oil		5.6	
Barley			20.2
Others^2^	4.4	4.4	4.4
Measured energy contents of diets, MJ/kg DM			
GE	17.93	19.08	17.77
AMEn	13.49	14.72	12.66
Measured GE of test ingredient, MJ/kg DM	–	39.37	17.99
Calculated energy value of test ingredient^3^, MJ/kg DM			
Hypothesis 1			
GE	–	39.37	17.99
AMEn 1	–	36.26	10.02
AMEn 2	–	36.26	10.02
Hypothesis 2			
GE	–	40.86	18.40
AMEn 1	–	37.76	10.44
AMEn 2	–	36.38	10.20
Hypothesis 3			
GE	–	37.85	17.57
AMEn 1	–	34.75	9.61
AMEn 2	–	36.15	9.84

AMEn = apparent metabolizable energy corrected for zero N balance.

1 From personal data and calculated according to the followiong 3 hypotheses. Hypothesis 1: GE of test diets 1 and 2 is adjusted for calculated GE of test ingredient equal to its measured GE. Hypothesis 2: GE of test diets 1 and 2 is 84 J/kg DM (i.e., 20 kcal/kg DM) higher than in hypothesis 1. Hypothesis 3: GE of test diets 1 and 2 is 84 J/kg DM (i.e., 20 kcal/kg DM) lower than in hypothesis 1.

2 Others is minerals and vitamins with zero GE content.

3 GE and AMEn 1 are calculated according to Eq. (1) in text; AMEn 2 is equal to measured GE of ingredient multiplied by calculated AMEn1/calculated GE (Eq. (4) in text).

As explained above, the ME value of complete feeds and the subsequent ME values of ingredients can be standardised according to the proportion of N in the feed that is retained in the body (or in eggs). In the case of poultry, the standardisation for zero N balance that is still frequently adopted generates ME values that are not dependent on the CP level and/or the amino acid balance of the B and T diets. However, that concept has not been used in pigs and should be progressively abandoned for poultry in order to propose standardised ME values (MEs) more representative of practical situations. This means that the standardisation of ME of B and T diets for a given level of N retention, say 50% of N intake, should be done before the calculation of ingredient ME values. Even the direct measurement of ME of cereals whose protein levels are rather low and amino acid profiles are imbalanced should also be standardised,. i.e., adding synthetic amino acids will improve the efficiency of N gain and provide a ME value close to the MEs value (Barzegar et al., 2019a). In the case of high protein ingredients included in a conventional B diet with a CP level meeting the requirements of growing pigs and birds, standardisation is highly important. Indeed, in the absence of standardisation, the measurement of ME of the T diet is accompanied by excessive catabolism and excretion of urinary N with a subsequently lowered ME value of the diet and the high protein ingredient. Unfortunately, most literature ME values for protein-rich ingredients for pigs and poultry have been proposed without any adjustment, presenting underestimated ME values.

The DE and ME values for pigs and poultry are measured on animals receiving either the B diet, or a T diet but not both of them and, in most literature papers, the energy value of the test ingredient is calculated for each measurement of the T diet, the difference being calculated between each T diet value and the mean value obtained on the B diet. That approach generates a number of observations per ingredient that is equal to the number of measurements of the T diet including that ingredient, allowing, in theory, the calculation of statistical indicators of accuracy or comparison of means. This method assumes that an animal (or a group of animals) receiving the T diet would have used the B diet as the mean of the animals receiving the B diet. This assumption is not acceptable, except that if the B diet and a T diet are measured on the same animal (or group of birds) under the same experimental conditions. The only acceptable solution consists in assuming that all the animals (groups of birds) receiving the T diet would use the B diet as another group of comparable animals, as far as the number of animals is high enough. Therefore, calculations are meaningful only on the average energy values per diet with no possibility for statistics.

In conclusion, the so-called difference method looks simple and basic. Unfortunately, the methods actually used are little documented, and more importantly, errors in design, measurements and calculations produce questionable energy values of ingredients. Such energy values can be troubling for the end-user who is not aware of these methodological considerations. Such a situation illustrates the inconsistency of the methods and calculations that may produce rather unreliable energy values for ingredients in published literature. Attention should therefore be paid to experimental and calculation approaches (when available) for using literature DE and ME values of feeds for monogastric.

### 6.2 Regression methods

One of the first regression methods is derived from the design used for the so-called difference method but with multiple levels of the test ingredient included in the B diet, with the MV inclusion level kept constant. A relationship between DCe or ME/GE of B and T diets and the percentage of inclusion of the test ingredient can then be calculated; its extrapolation to a full replacement of the B diet by the test ingredient provides an estimate of DCe or ME/DE of the test ingredient (Adeola, 2001). A frequent error applied in this approach is the extrapolation to 100% while it should be extrapolated to 100% minus MV%. In addition, as for the difference method, the percentage of the test ingredient is expressed on an ”as-fed” basis instead of on a DM basis. For these above reasons as well as because of the high number of diets and animals to be used (and associated costs, etc.), this method is used infrequently.

The second regression method involves the measurement of DE or ME of complex diets containing several ingredients at variable inclusion levels, the coefficients of correlation between the levels of inclusion of ingredients being as low as possible and, if possible, mostly close to zero. According to that design, the GE, DE or ME value of the complex diet is a combination of the GE, DE or ME independent contributions of each ingredient which follows a linear multiple regression model as:

GE = a_1_*x*_1_ + a_2_*x*_2_ + · ·  · + a_*n*_*x*_*n*_ ,

DE = b_1_*x*_1_ + b_2_*x*_2_ + · · · + b_*n*_*x*_*n*_ ,

ME = c_1_*x*_1_ + c_2_*x*_2_ + · · · + c_*n*_*x*_*n*_ ,

where GE, DE or ME are the measured GE, DE or ME values of the complex diets; *x*_1_, *x*_2_, … *x*_*n*_ represent the percentages units of *n* individual ingredients included in the diet; and a_1_, a_2_, … a_*n*_, b_1_, b_2_, … b_*n*_, and c_1_, c_2_, … c*_n_* denote the estimated GE or DE or ME values of the *n* ingredients which correspond to the calculated coefficients in the regression equations.

All energy values are expressed on DM and percentage units as DM per DM; the MV should also be kept constant in all diets and is considered as not providing any energy. Statistically, the calculation of the regression model is possible if the number of measured diets is higher (at least n + 1) than the number of ingredients. As indicated for the difference method, the GE, DE or ME estimates of each ingredient from the multiple regression model correspond to GE_0_, DE_0_ and ME_0_ of each ingredient. GE_0_ of an ingredient is usually different from the measured GE of that ingredient in a bomb calorimeter, and the difference will have a direct and mathematical impact on DE_0_ or ME_0_. Therefore, the GE of the ingredients should be analysed in the laboratory. The DCe or ME/DE or ME/GE of ingredient i are therefore calculated as the ratios bi/ai, ci/bi or ci/ai, respectively; and the final DE or ME values of ingredient i are obtained as measured GE of this ingredient multiplied by DCe or ME/GE, respectively. To date, this method has been used in pigs (Cozannet et al., 2012; Noblet et al., 1993a; van Milgen et al., 2001) but rarely in poultry (Barzegar et al., 2019a). Its main advantage is that it allows the preparation of conventional diets that meet the animal's requirements with ingredient inclusion levels comparable to practical use. Although it has not been widely recognised, it also offers the possibility of evaluating interactions between ingredients if the design is appropriate in terms of the number of diets and levels of inclusions. As detailed later, these advantages are more pronounced in the case of NE evaluation of ingredients. Finally, as for the other evaluation methods for poultry, the measured ME value of the diets should be adjusted as AMEn or AMEs in order to calculate the AMEn or AMEs values of each ingredient; in the case of pigs where the AMEs concept is more frequently used, the ME of diets should also be adjusted to AMEs. Otherwise, if calculated from measured ME, the regression model will provide AME values that should be rather close to the AMEs values since the complex diets should be well balanced for amino acid levels. Caution must be made when this approach is used in practice as any experimental, human or laboratory mistakes can affect all estimated energy values of the ingredients involved because all the calculations rely on to each other in the regression process.

### 6.3 Other methods

Apart from direct in vivo measurements of DE or ME in pigs or in poultry, the energy value of ingredients can be estimated in vitro by simulating the different steps of digestion in “test-tubes”. The methods are similar to those described for complete diets, and a relationship between the in vitro and in vivo digestibility values is required for estimating the “true” energy value of the ingredient. This relationship is usually better when the values are obtained and applied for a homogenous group of ingredients (Regmi et al., 2008, for barley, for instance). Otherwise, in vitro values alone may be used for ranking feeds without any reliable information on the actual energy value of the ingredient (Jaworski et al., 2015). As for diets, the in vitro approach is unable to detect the effect of age of the animal, feed manufacturing technology, and feed additives.

The DE and ME value of ingredients can also be calculated from chemical indicators or from their NIR spectra, both approaches being precise and reliable when they are applied to a group of homogenous ingredients (cereals and their by-products, for instance, van Barneveld et al., 1999; Zijlstra et al., 2011) or, preferably, to a single ingredient whose chemical composition may vary widely (wheat and its by-products or animal by-products; for instance, Li et al., 2016). The prediction equations from chemical composition or NIR spectra must be established, and the in vivo measurements of DE and ME on which the calibration is based should be obtained under standardised methods. While most equations based on chemical indicators are published, the NIR prediction is based on proprietary calibrations obtained using in vivo measurements specific to the owners of the NIR spectroscopy machines and hence are often not put out in the public domain.

The NIR prediction is rapid, cheap, convenient, precise and accurate as long as it is obtained per group of homogenous feedstuffs. It is routinely used, especially for poultry AME values. But, as stated above, the domain of validity and applicability of the NIR prediction should be clearly stated (AMEn and not AME; AMEn for broiler or adult rooster; DE for growing pig; for mash and not pellet; etc.). It should also be noted that the accuracy of NIR or chemical estimates of the ME values is very much dependent on the accuracy of bioassays. As indicated earlier, any potential flaws must be avoided for the establishment of the NIR calibration curve or chemical prediction equation. Finally, one frequent mistake in using prediction equations based on chemical composition relates to applying general equations obtained on a large set of diets (Le Goff and Noblet, 2001) to a specific ingredient where there are discrepancies due, for instance, to the composition and digestion of its dietary fibre fraction and the absence of effect of the minerals originating from the MV fraction of the diet (see above Table 2).

## 7 Net energy values of feeds in pigs and poultry

Net energy is a rather old concept (Armsby and Fries, 1915) that has been used in domestic animals, rodents and humans. It is mostly based on the development of calorimetry methods, either direct or, much more commonly, indirect techniques (Brouwer, 1965; McLean and Tobin, 1987). With regard to domestic animals, a huge piece of work was done on poultry, pigs and ruminants at Rostock Station in former East Germany with the publication of Schiemann et al. (1972) as its major outcome and the subsequent application of the concept in pigs and ruminants. The development and the interest in NE over the last 70 years have also been quite variable according to the animal species: intense for ruminants (especially the dairy cow), relatively minor for poultry (Emmans, 1994; Fraps, 1946) and intermediate for pigs (Just, 1982; Noblet et al., 1994a). These variations in interest and available scientific knowledge are reflected in the use of NE in the different animal production sectors: high for ruminants, negligible for poultry and intermediate for pigs. However, in the case of poultry, a few major publications (Carré et al., 2014; Cerrate et al., 2019; Wu et al., 2019) and heightened interest by the poultry industry sector have been noticed over the last 10 to 15 years with a potentially greater application of the NE concept within the next ten years.

### 7.1 Definitions

**Fig. 1:**
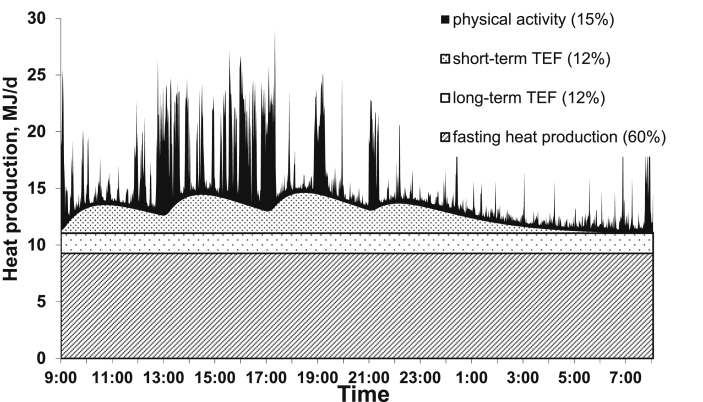
Components of heat production in a growing pig (60 kg) offered 2.4 MJ ME/kg BW^0.60^ per day in 4 meals at 09:00, 13:00, 17:00, and 21:00. TEF = thermic effect of feeding; from Noblet and van Milgen (2013).

Net energy (NE) is defined as the ME content minus the heat increment (HI) associated with feed utilisation (i.e., the energy cost of ingestion, digestion, and metabolic utilisation of energy) and the energy cost corresponding to a “normal” level of physical activity (Fig. 1). The NE-to-ME ratio (or k) corresponds to the efficiency of ME utilisation for NE; it also corresponds to 1 − (HI/ME). However, the HI/ME ratio of a given feed depends on the ME intake level as well as on several animal and environmental factors. For instance, the HI is lower for ME supplied below the maintenance energy requirement than for ME supplied above maintenance energy requirement (Noblet et al., 1993a, 1994a, 1994b). But it should be noted that the HI for meeting the requirements for maintenance is an “apparent” HI equal to the difference between hear production (HP) of feed and HP corresponding to the mobilisation of body reserves (i.e. fat) under fasting. The HI is also lower when ME is used for fat deposition than for protein deposition (90% vs. 60% in pigs according to Noblet et al., 1999). As fat deposition typically increases more rapidly than protein deposition with increasing ME intake, HI/ME should, at least theoretically, be lower at higher levels of ME intake.

In most production conditions, ME intake is used for meeting the requirements for maintenance and those for the production of weight gain (growing pig, pregnant sow, broiler) or eggs (laying hen) or milk (lactating sow). Therefore, it is not possible to differentiate the metabolic utilisation of feed ME between these different functions on a given animal. This means that HI/ME or k must be obtained for the combined utilisation of ME for maintenance and production. The practical consequence is that for maintaining the concept of a single NE value for a given feed or raw material, it is necessary to determine the NE value under standardised nutritional and animal conditions: at protein and amino acid supplies meeting the requirements, a constant composition of the gain, a standard level of performance, and at a given physiological stage. Only under such conditions, is it possible to compare the NE values of different feeds. In other terms, the comparison of NE values of different feeds obtained under quite different animal and environmental conditions is, in theory, not possible.

In practice, variable physiological situations exist in both pigs and poultry: growing pigs and broilers (maintenance + high rate of energy gain as BW), pregnant sows (maintenance + low rate of energy gain), lactating sows (maintenance + very high export of energy as milk) or laying hens (maintenance + high export of energy in eggs). This means that, in theory, there should be as many NE values of feeds as different physiological situations within a species; however, if HI/ME values in a series of feeds are ranked similarly for growing pigs, pregnant sows and lactating sows, for instance, the NE system developed for one stage of production can be applied to the other stages. That will be detailed later.

Measuring NE of feeds responds to 3 main objectives: 1) quantification of NE value of a feed under variable animal (effect of BW or genotype, for instance) and environment conditions (ambient temperature, for instance); 2) estimation of the effect of manufacturing technologies and feed additives applied to a diet or a series of diets with a common methodological approach, and 3) building a database with NE values of different feeds obtained under the same animal and environment conditions, with the same methodologies in order to calculate relationships between characteristics of the diets and their NE values. For the latter, linear regression models between dietary NE and some predictors are produced, the predictors being the digestible nutrient contents, the DE or ME values plus some chemical indicators (Noblet et al., 1994a; Wu et al., 2019), and the chemical characteristics or the NIR spectra of the diet. Some of these equations are presented in Table 5. One equation and, preferably, a group of equations, established from the same database with different regression models define what is termed an NE system (Noblet et al., 1994a; Wu et al., 2019). It should also be noted that some NE prediction equations, and hence NE systems, do not arise directly from a data set obtained using strictly controlled experiments but from a compilation of literature equations (CVB, 2018) and/or biochemical and in vitro indicators (Boisen and Verstegen, 1998). In practice, such an equation is not easy to develop because of the errors that occur in each disparate piece of studies published in the literature due to the different experimental conditions, such as the age and breed of animals, ingredients used in the diets, and the husbandry conditions of the facilities, just to name a few. However, should a robust and reliable NE system be developed, then it would provide tremendous advantages because such an equation can be applied to any feed (complete diet or ingredient) as far as the predictors used in the equations are robust and available, allowing an NE system to be implemented in the feed industry without any use of the sophisticated equipment and methodologies for measuring NE.

**Table 5:** NE prediction equations for pigs and poultry.

Equation^1^	Source^2^
Growing pigs	
NE = 0.121 DCP + 0.350 DEE + 0.143 ST + 0.119 SU + 0.086 DRes	1
NE = 0.703 DE - 0.041 CP + 0.066 EE - 0.041 CF + 0.020 ST	1
NE = 0.700 DE – 0.038 CP + 0.067 EE – 0.037 ADF +0.020 ST	1
NE = 0.117 DCP + 0.357 DEE + 0.141 (ST + GOS + 0.90 SU) + 0.097 FCH + 0.106 AC + 0.146 PR + 0.195 BU + 0.207 ETH + 0.120 LA + 0.138 GLYCEROL	2
Broilers	
NE = 0.781 ME - 0.028 CP + 0.029 EE	3

1 CP: crude protein, EE: ether extract, ST: starch, SU: sugars, DCP: digestible CP, DEE: digestible EE, DRes: digestible residue (i.e., difference between digestible organic matter and other digestible nutrients considered in the equation); GOS: sugars, glucose and oligosaccahrides, FCH: fermented degradable carbohydrates, AC: acetic acid, PR: propionic acid, BU: butyric acid, ETH: ethanol, LA: lactic acid; some specifications of the analytical methods used for nutrients are indicated in CVB (2018) for the CVB equation. NE, DE or ME as MJ/kg DM and nutrients as percentages of DM.

2 1: Noblet et al. (1994a); 2: CVB (2018); 3: Wu et al. (2019).

In conclusion, the NE value of a diet is dependent on the animal and environmental conditions for its measurement. Similarly, the NE value of a diet or an ingredient is highly dependent on the NE system or the equation that is used for its calculation. Fortunately, as detailed later, the number of available and validated systems per animal species is rather limited, and the most important ones are quite consistent (CVB, 2018; Noblet et al., 1994a). Finally, the routine measurement of NE values of diets and ingredients is costly and time-consuming as it requires specific expertise. Hence routine measurements of NE values are therefore not really justified. The simple application of an available and validated NE prediction equation based on digestibility predictors obtained from well-controlled animal experiments is usually sufficient for implementing NE values for feed formulation.

### 7.2 Methods for NE measurement in pigs and poultry

As shown in Fig. 1, HI is equal to Total HP minus Fasting HP (or FHP); as NE is equal to ME intake minus HI, NE is then equal to ME – (HP – FHP) or (ME – HP) + FHP; since ME – HP represents the energy gain (RE; in BW or as milk or as eggs), NE in producing animals is calculated as RE plus FHP. As indicated below, RE can be estimated directly according to the comparative slaughter technique (CST) or calculated as the difference between ME intake and HP. The measurements or estimations of RE or HP and FHP are thus necessary for evaluating the NE content of a feed; the concomitant measurement of DE or ME intakes according to the methods described above are also required (Noblet et al., 1994a).

#### 7.2.1 Measurement of energy gain: comparative slaughter technique

The direct measurement of energy gain over a given experimental period in growing animals can be evaluated as the difference between the energy content measured in BW at the end of the experiment minus the energy content in BW at the start of the experiment. In the case of animals producing egg or milk, the energy gain corresponds to the exported energy in eggs or milk corrected for the changes in body energy content. The initial body energy content of the experimental animals is evaluated from contemporary and similar animals measured for their body energy content at the beginning of the trial. In most cases, the body energy content is measured after slaughter, grinding of the total body, conditioning of a representative sample of total body and gross energy measurement in pigs (Just, 1982; Kil et al., 2011) and poultry (Barekatain et al., 2014; Carré et al., 2014; Moscoso-Muñoz et al., 2020). The energy content in the body can also be estimated on live animals by scanning methods such as the DEXA method used by Cerrate et al. (2019) in broilers. The advantage of the scanning methods is that no slaughtering and tedious grinding, and homogenisation are required, and the same animals can be measured at the beginning and end of the experiment. However, its accuracy is compromised due to the errors associated with the instrument, the in-built mathematical estimates, and the scanning of live animals. Overall, the CST method used in both small (fish, poultry) and large animals (pigs, ruminants) requires a sufficiently long experimental period in order to amass enough energy content in the body over the trial to attenuate the potential errors of the estimation of initial body composition. With this approach, each animal can be measured only once (except if scanning methods are used), and the response obtained may correspond to different successive physiological situations and therefore represents a combined response if the experimental period is rather long. Finally, each experimental animal must be kept under well-controlled environmental conditions for its thermoregulation, behaviour and health in order to minimise and standardise the energy requirements for maintenance. In conclusion, the CST remains popular since it does not require any sophisticated equipment, but it is laborious and the response does not provide any dynamic response of the animals either over a nychthemeral period or over a longer period and, more importantly, over successive periods on the same animal.

#### 7.2.2 Measurement of heat production: indirect calorimetry methods

Heat production can be measured directly through direct calorimetry with, for instance, a gradient layer calorimeter used mainly in the UK in the 1960s to 1980s (Close and Mount, 1975) or, more commonly, estimated from indirect calorimetry through the measurement of oxygen consumption and carbon dioxide production in respiration chambers. The principle is that the complete combustion of organic matter is accompanied by the consumption of O_2_ (VO_2_) and the production of CO_2_ (VCO_2_) with the release of resulting heat (HP). The combination of combustion of nutrients and application of corrections related to incomplete oxidation of nutrients, for instance, CH_4_ (VCH_4_) and H_2_ release and to urinary N (Nu) excretion, allows the calculation of HP owing to VO_2_, VCO_2_, VCH_4_ and Nu. The most accepted equation for farm animals was proposed by Brouwer (1965):

HP (kJ) = 16.175 × VO_2_ + 5.021 × VCO_2_ – 5.98 × Nu – 2.167 × VCH_4_ ,

where VO_2_, VCO_2_, VCH_4_ are in litres and Nu in grams.

The indirect calorimetry methods are based on either 1) the continuous measurement of the difference in O_2_ and CO_2_ contents between the ingoing air and the outgoing air multiplied by the air flow rate through the respiration chamber in the open circuit system (van Milgen et al., 1997) or 2) the consumption of O_2_ provided by an O_2_ cylinder and the production of CO_2_ entrapped in a train in the closed-circuit system (Wu et al., 2019). For the latter, the weights of O_2_ and CO_2_ over a period of time, 24 h, for instance, are determined and used for the calculation of HP. The closed-circuit system can be used for small size animals over short durations (<24 h). But it is more laborious than the open-circuit system and does not allow any observation of the dynamics of HP over the measurement period. On the other hand, the system is more reliable and accurate for the measurements of VO_2_ and VCO_2_, being gravimetric and chemical, respectively. Unlike the CST method, the indirect calorimetry systems allow measurements over short periods of time (i.e., 1 to 5 d) with possibilities of successive measurements under different feeding, housing and physiological conditions on the same animal(s). In the open-circuit system that can be fully automatised, the dynamics of HP over very short periods (<1 h) can be evaluated (Kuhla et al., 2015) and used in modelling approaches to partition the total daily HP between different components, which can be used in the further interpretation of energy balance data or correction for differences in the level of physical activity (Labussière et al., 2013, 2015; van Milgen et al., 1997) (Fig. 1). Finally, the indirect calorimetry system can also allow the concomitant use of metabolic tracers to examine specific metabolism (protein) of substrates (Van den Borne et al., 2015). The full details, constraints of the measurements and the calculations for implementing the indirect calorimetry methods have been described by Gerrits et al. (2015) and Alferink et al. (2015).

**Fig. 2:**
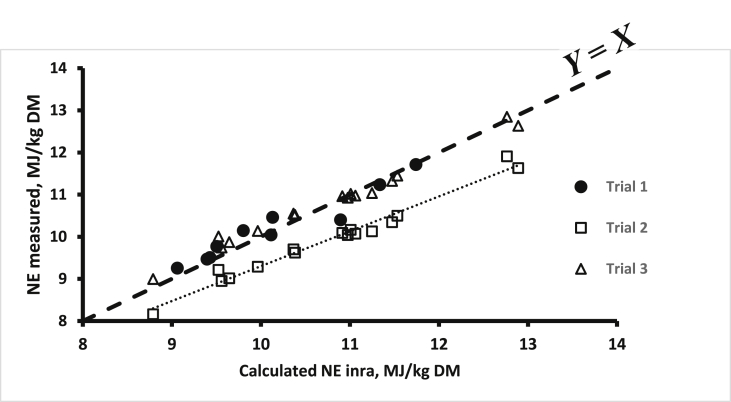
Relationship between measured NE of 26 pig diets (Institute for Livestock Feeding and Nutrition Research, personal communication; FHP = 750 kJ/kg BW^0.60^) and their NE value calculated according to Eqs. (Eq. 2), (Eq. 4)) (mean of both) of Noblet et al. (1994a); trial 1 (*n* = 10 diets) and trial 2 (*n* = 16 diets) correspond to values of 2 successive series of measurements; the measured and calculated values are almost identical for trial 1 (10.20 and 10.14 MJ/kg DM) but markedly different (9.93 and 10.76 MJ/kg DM) for trial 2; trial 3 data correspond to values of trial 2 when HP is equal to 0.905 measured HP (see text for explanations). NE = net energy; FHP = fasting heat production; HP = heat production.

The open-circuit indirect calorimetry system is more popular than the closed-circuit calorimetry, even for small-sized animals like broilers. However, one major concern for this methodology is the calibration of the system, especially with regard to VO_2_ measurements. Indeed, VO_2_ is a major contributor in the calculation of HP (about 3 times more than VCO_2_) and, in connection with differences in the technologies used for measuring O_2_ (based on its paramagnetic properties; possible effects of relative humidity and barometric pressure) and CO_2_ (based on infrared equipment), the concentrations of O_2_ in the air are more difficult and complex to measure than that of CO_2_. In addition, the CO_2_ concentrations range from close to 0% in the ingoing air to a maximum of 1% in the outgoing air. The full range of measurement of a 0% to 1% gas analyser can then be used, and the accuracy of the measurement is quite satisfactory. On the other hand, O_2_ concentrations range from about 21% in the ingoing air to a minimum of 20% in the outgoing air. If calibrated between 0 and 21%, only a very small part of the 0% to 21% measurement range is used with a subsequent low accuracy of O_2_ content that is further accentuated for the calculated difference in O_2_ content between the ingoing and the outgoing air. The alternative solution is to use differential O_2_ gas analysers for which the baseline is not 0% O_2_ but the O_2_ content of the atmospheric air (i.e., ingoing air) (van Milgen et al., 1997). Overall, great attention should be paid to the daily calibration of the gas analysers, especially for O_2_, as well as the gas meters in order to obtain reliable VO_2_ values. The potential impact of wrong calibrations and uncontrolled deviations of the gas meters and gas analysers is illustrated in Fig. 2. It shows the relationship between predicted NE values of 26 compound feeds and their measured NE values (4 replicates per diet; 35 to 100 kg pigs) in successive series of measurements over 1981 to 1983 in the same experimental facilities in the Netherlands (Institute for Livestock Feeding and Nutrition Research, IVVO internal reports 91, 107, 138 and 150, personal communication). Clearly, 2 groups of measurements can be identified, one with a good correspondence between calculated and measured values (+0.6%) and one with a significant difference (−7.7%). This difference suggests an underestimation of the dietary NE value due probably to an overestimation of measured HP and associated gas exchanges and a subsequent underestimation of RE. This would correspond to a 9.5% underestimation of the HP.

The measurement of VO_2_ and VCO_2_ allows the calculation of respiratory quotient (RQ) defined as VCO_2_/VO_2_. This ratio varies with the feeding level being minimal (#0.70) in fasted animals catabolising body fat and maximum (#1.10) in ad libitum fed animals. The RQ also varies with the energy-yielding nutrients provided by the diet, i.e., lower when the dietary CP and fat contents are high and higher when the starch level is high (van Milgen et al., 2001). This criterion is then specific to a given nutritional situation and a given animal model. In addition, it is reasonably constant over the successive days of an energy balance period. This means that RQ can be used as a reliable indicator of VO_2_ and VCO_2_ measurements, especially for detecting errors in VO_2_ measurements.

Energy gain in BW or as milk or as eggs can be obtained directly using the CST method and indirectly using the difference between ME intake and HP, whereas HP is measured directly in the direct calorimetry methods and indirectly from gaseous exchanges (HPcal). These methods have advantages and disadvantages as well as different domains of application. Furthermore, HP and RE obtained using various methods have been compared (Gerrits et al., 2015). First, direct and indirect calorimetry techniques when they are run simultaneously would produce comparable estimates of HP in humans (Dauncey, 1980); but no data are available in pigs and poultry. Second, the CST method would provide RE values slightly lower than when obtained as the difference between ME intake and HPcal. With continuous HPcal measurements over the 6 weeks of a CST experimental period, Quiniou et al. (1995) obtained a 4% difference in RE. If conducted on 2 different groups of animals, the comparison of the CST and HPcal methods may have been biased by the environmental conditions, the associated changes in behaviour and thermoregulatory demand that may differ between the respiration chambers and the housing conditions. Under these conditions, energy expenditure is expected to be systematically higher and RE lower with the CST method when adjusted for comparable ME intakes.

#### 7.2.3 Fasting heat production in pigs and poultry

**Fig. 3:**
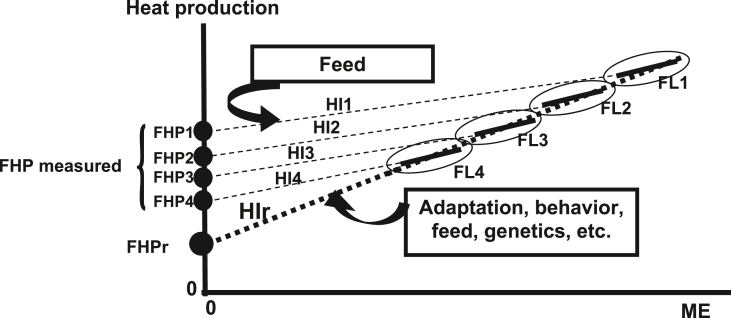
Schematic representation of the effect of feeding level (FLi) on heat production and fasting heat production (FHP) in nonruminant animals. Each FHPi corresponds to the FHP measured on animals receiving the FLi during the immediately preceding period. The FHPr (*r* for regression) is obtained from the regression between hear production (HP) and metabolisable energy (ME). The slope is the “regression” heat increment (HIr), and the slope between each FHPi and HPi corresponds to the measured heat increment (HIi) (from Noblet and van Milgen, 2013).

As indicated earlier, an estimate of FHP is required for calculating NE values. The FHP of animals can be obtained by measuring HP at variable ME intakes and calculating a regression between HP and ME intake that is extrapolated at zero ME intake (Fig. 3; FHPr). For that regression methodology, the HP can be measured by calorimetry or calculated as the difference between ME intake and energy gain, energy gain being obtained according to the CST method (see above). This regression method was well used in the past and has very important limitations. First, it extrapolates HP measured at feed intake levels, typically between 65% and 100% of ad libitum intake, to HP at zero feed intake. When the regression method takes in HP values obtained far outside the measurement range, inaccuracies in the estimated intercept become apparent. Second and more importantly, the measured FHP is as low as the feeding level prior to fasting is low, especially in growing animals (de Lange et al., 2006b; Koong et al., 1982; Labussière et al., 2011). This can be interpreted as an adaptation of the animal to a relative undernutrition diet leading to a subsequently reduced total HP. The variation in HP with reduced ME intake (and the associated slope) is, therefore, due to both a reduced HI related to reduced ME intake and a reduced basal metabolic rate of the animal. The consequence is that FHPr is markedly lower than the measured FHP with subsequent lower values for NE and k, and a higher HI (Fig. 3). These authors also observed that HI, calculated as HP minus the measured FHP and expressed as per unit of ME intake, is constant for the different feeding levels. Furthermore, the degree of adaptation of FHP and HP to feeding level also depends on animal characteristics such as the genotype (Barea et al., 2010; Renaudeau et al., 2006) with FHPr values being quite low and even negative. Sometimes it is highly variable and not consistent between locations (Kil, 2008). Overall, these observations suggest that FHPr represents a wrong concept for the basal metabolic rate of farm animals that should not be used anymore as an estimate of FHP in the calculation of NE values. Measured FHP in animals fed at near *ad libitum* level is highly preferable (Labussière et al., 2011).

The measurement of FHP immediately after a feeding period can be carried out by direct calorimetry (Close and Mount, 1975), but over the last 20 to 30 years, mostly indirect calorimetry methods have been used, whatever the size of the animal. After feed withdrawal, the HP of the animal decreases regularly and reaches a plateau value after a minimum of 15 h that corresponds to complete digestion and metabolism of consumed nutrients. Feed withdrawal may also generate an excessive and variable physical activity leading to variability in total HP over the fasting period. Finally, under an underfed or starving condition, the lower critical temperature (LCT) is increased with a potential increase in HP for thermoregulation of the animal after a prolonged fast (Close and Mount, 1975). Therefore, most FHP measurements in pigs and poultry result from HP data obtained over 18 to 24 h after feed withdrawal. The animals are kept in the dark to reduce their physical activity and at ambient temperatures above recommended LCT of fasted animals (Li et al., 2017, 2018a); the HP is then minimal and considered as an estimate of FHP. A more sophisticated method proposed by van Milgen et al. (1997) consists of modelling the decrease of HP and the contribution of physical activity HP over 24 h after feed withdrawal. The asymptotic HP is assumed to correspond to FHP at zero physical activity. The FHP values thus obtained (Barea et al., 2010; Noblet et al., 2015) do not include any activity HP and are lower but close to those obtained over a few hours after a minimum 18 h fast at very low levels of physical activity (dark). A longer fasting period is not justified due to potential animal welfare concerns, continued reduction of HP due to a prolonging of the fast, and subsequent lowered FHP values not representative of the animal in its previous nutritional situation (Close and Mount, 1975).

A modified regression method based on successive measurements of HP on the same animal (or group of animals) at 2 feeding levels (100% for 5 d followed by 60% for 2 d) has also been used in pigs for the calculation of FHP from the linear relationship between HP and ME intake (Liu et al., 2014; Noblet et al., 1994a). Liu et al. (2014) demonstrated that the HP on the 2nd d at a low feeding level allowed the pig to rely on its new feed intake but not to adapt its metabolism to the undernutrition condition with a further reduction of HP over d 3 and 4 on the low feeding level (as observed in animals restricted for long periods). The FHP obtained with that methodology (Noblet et al., 1994a) was identical to the FHP values obtained later in the same facilities (Barea et al., 2010; Le Bellego et al., 2001; van Milgen et al., 2001) from a direct measurement of FHP.

The compilation of literature data on FHP values for several adult animal species ranging in size from the mouse to the elephant indicated that the 0.75 interspecies coefficient could be used for calculating the so-called metabolic BW (kg) calculated as BW^0.75^, which allowed a constancy of FHP over a large range of BW in adult animals within a species or even between animal species and humans (Bowes et al., 2021; Brody, 1945; Kleiber, 1961); FHP values and associated ME requirements for maintenance (MEm) were then expressed per kg BW^0.75^. This concept was used for growing animals, despite new experimental data in growing pigs (Koong et al., 1982; Tess et al., 1984) suggested that FHP was not proportional to metabolic BW (BW^0.75^). These results were confirmed later in pigs (Noblet et al., 1999) and broilers (Noblet et al., 2015), with the most appropriate exponents being 0.60 in growing pigs and 0.70 in broilers. The recommended FHP values for growing pigs and broilers are thus about 750 kJ/kg BW^0.60^ and 450 kJ/kg BW^0.70^, respectively.

In conclusion, the oldest literature values on growing animals expressed per kilogram BW^0.75^ and usually obtained according to the regression method applied to different groups of animals fed different feeding levels should be abandoned and replaced by more recent FHP values obtained from directly measured FHP values at a minimal level of physical activity and at thermoneutrality for which the best exponent is species specific (Labussière et al., 2011).

#### 7.2.4 Practical considerations for NE measurement of feeds for pigs and poultry

NE of a given quantity of daily feed to an animal or a group of animals is calculated as the addition of their daily RE and their daily FHP, with the latter quantity corresponding to the average metabolic body size over the fed period multiplied by the FHP per kilogram of metabolic body weight (FHP∗). Daily data are further expressed as per kg of daily feed DM intake (Noblet et al., 1994a, 1994b). If the indirect calorimetry methods are used, the FHP∗ can be measured on each animal (or each group of animals) but, as explained above, FHP is obtained over a very short duration (i.e., a few hours) and potentially rather inaccurate and variable between animals due to insufficient accuracy of the measurement technique as well as the impact of physical activity that is not quantified in most situations. It is, therefore, preferable to use a common FHP∗ value as the mean of all individual FHP∗ values within a trial (Noblet et al., 1994a) or simply use a literature FHP∗ value if no FHP∗ values are available (Wu et al., 2019). As illustrated in Fig. 1, HP varies over each day with the occurrence of meals and the behaviour of the animal(s). This means that to get a correct evaluation of the daily HP by calorimetry methods which corresponds to the actually measured ME intake, HP should be measured continuously over the 24-h period. Particular attention should be paid to the periods during and just after the meals, which associate with peaks of HP whose amplitude may be quite variable between animals. In other words, the shorter the pause periods of measurement of HP are during the day, the more representative the measured daily HP will be. In practice, the pause period for calibrating the system, taking care of the animals and so on should not exceed 1 h/d and should not include any meal. Daily HP may also vary between successive days of measurement in regards to growth of the animal, naturally variable daily feed intake and a need for adaptation of the animal to the respiration chamber environment. More specifically, if no adaptation in a comparable environment is conducted beforehand, the 1st d in the respiration chamber data should be ignored as it won't obtain a representative estimate of daily HP. A minimum of 2 full days of HP measurement would be required in growing pigs and broilers. Longer measurement periods (up to 5 d) are suggested in heavier or reproductive animals (laying hens, sows). Unfortunately, some studies failed to follow such experimental conditions (Kim et al., 2020).

The measurement or calculation of RE using either the CST or HPcal method are considered to be reliable. But FHP∗ is not easy to measure precisely, and, in the case of the CST method, it is impossible to estimate directly or by regression. In addition, the housing conditions for animals used for the CST method that may have been detrimental, i.e., variable ambient temperatures and excessive physical activity pushing up FHP∗, is ignored in the NE calculations since only the literature FHP values obtained under favourable experimental conditions can be used. This situation explains the lower NE as well as k values (i.e., NE/ME) obtained by Just (1982), and, more recently, by Kil (2008) in growing pigs with the CST method than in the studies of Noblet et al. (1994a) by indirect calorimetry. On the other hand, Carré et al. (2014) with the CST method and Wu et al. (2019) with the HPcal method used broilers housed under favourable conditions and using the same literature bases for estimation of FHP∗ (Noblet et al., 2015) obtained similar efficiencies while the NE or k values proposed in broilers by Cerrate et al. (2019), who used the CST method and applied a very low literature FHP value, are lower. In conclusion, FHP∗ estimate has an important impact on NE values obtained using poorly controlled experiments or inappropriate calculations. One illustration of the impact of inadequate use of FHP∗ is given by Jaworski et al. (2016) who obtained k values of 3 diets averaging 50% in growing pigs while a recalculation from their published energy balance data (ME intake, RE, HP) produces k values averaging 78%, with these latter values being consistent with literature values for similar diets or with values obtained in the same research unit (Li et al., 2017). As a guideline, the k value of diets should range between 70% and 80% in growing pigs and broilers (Noblet et al., 1994a; van Milgen et al., 2001; Wu et al., 2019).

In conclusion, the NE value of a feed and the corresponding k value should be evaluated according to standardized and proper methods. For growing animals, it is suggested to use energy balance measurements in similar animals (i.e., same sex, same breed, and in the same body-weight range), keep these animals within their thermoneutral zone, minimize variations in behaviour, and feed the animals at about the same feed intake level with balanced diets so that the animals can express their growth potential. Under these circumstances, HP and NE data will be related only to dietary effects and an erroneous estimate of FHP will affect the absolute NE value, but not the ranking between feeds. Finally, since the absolute NE values are dependent on assumptions and calculations methods (FHP), conditions of measurement (e.g., climate, activity) and the composition of the energy gain, the data on NE and k available in the literature for pigs and poultry should be interpreted with caution and may not be directly comparable across studies or between feeding tables.

### 7.3 Validation of NE systems

As described above, a NE system is materialized by a prediction equation or a model originating from the compilation of NE measurements conducted under controlled and optimal conditions. Additionally, the design of the experiments in terms of variability and independency of chemical characteristics of diets should allow the calculation of robust regression equations (Barzegar et al., 2019b; Noblet et al., 1994a; Wu et al., 2019). The equation can also originate from an empirical combination of several regression equations with additional prediction criteria. The latter type of equations is illustrated by the successive NE equations for pigs published by the CVB in The Netherlands, which, in turn, evolved from the equation of Schiemann et al. (1972), to the reanalysis of the data of Noblet et al. (1994a) with additional criteria such as the lactic acid and short-chain fatty acids levels in the last CVB (2018) Tables. One consequence is that the NE values predicted according to the equations of Noblet et al. (1994a) and CVB (2018) are almost identical. However, CVB (2018) equation requires additional criteria, values of which may suffer from lack of documentation and justification.

However, before being applied in practice, an equation or a model must be evaluated and validated. For NE prediction equations, 2 approaches can be used: 1) the comparison of measured NE values of feeds and their NE values calculated according to that model, and 2) the performance response of animals such as the energy cost of BW gain or egg production according to the energy evaluation model (DE vs ME vs NE). The applicability of NE equations usually established from NE measurements conducted on diets should also be validated for evaluating the NE of ingredients (Noblet and van Milgen, 2004).

**Fig. 4:**
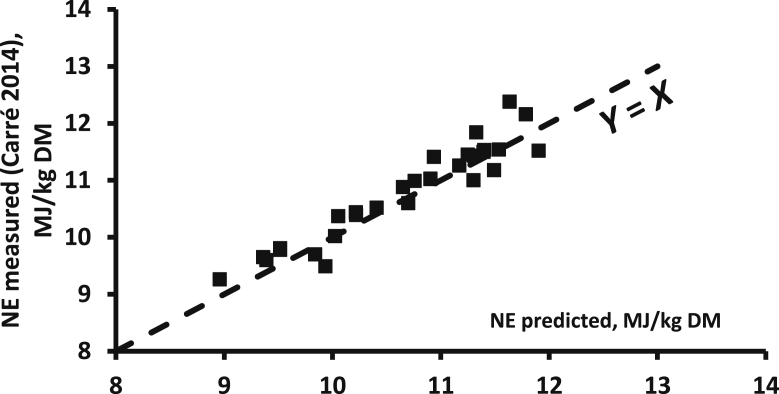
Relationship between measured NE of 29 broilers diets (Carré et al., 2014; mean = 10.79 MJ/kg DM; FHP = 500 kJ/kg BW^0.60^) and NE values calculated according to Wu et al. (2019) (mean = 10.66 MJ/kg DM; FHP = 450 kJ/kg BW^0.70^); the correlation coefficient between the 2 sets of values is 0.95. NE = net energy; FHP = fasting heat production.

For poultry feeds, 3 groups of NE equations have been proposed recently in broilers between 21 and 35 d fed 29 different diets (Carré et al., 2014) using the CST method, in broilers between 1 and 21 d fed 10 different diets (Cerrate et al., 2019) using the CST method, and in 24 to 28 d broilers fed 19 different diets (Wu et al., 2019) using the indirect calorimetry method. When considering a common indicator such as the variations of k (or NE/AME) with the chemical composition of diets, all 3 equations indicate a positive effect of fat content and a negative effect of protein content on k, the intercept corresponding approximately to the k value for starch energy. These results are also confirmed in smaller-scale trials by Noblet (2015), Liu et al. (2017) and Moscoso-Muñoz et al. (2020). This means that, as for pigs (Noblet et al., 1994a), the efficiencies of ME are the highest for fat energy and the lowest for protein energy and intermediary for starch energy. However, the absolute values of k (as NE/AMEn) differ between the 3 main studies: 0.800 for Carré et al. (2014), 0.724 for Cerrate et al. (2019) and 0.789 for Wu et al. (2019). The 3 studies differ slightly for their average chemical characteristics but the differences do not explain the markedly lower k value in the study of Cerrate et al. (2019) that might be related to the younger age of the birds but also to the methodology based on the CST method and probably a very low value set for the FHP (see above). The k values are remarkably close for the 2 other studies conducted in older birds with comparable FHP values; the similarity of the 2 studies is also illustrated in Fig. 4 that represents the relationship between the NE values measured in the study of Carré et al. (2014) and the NE values of the same diets calculated according to the equations proposed by Wu et al. (2019). However, both the k prediction and the NE prediction equations indicate a higher negative effect of protein and a higher positive effect of fat in the study of Wu et al. (2019), with the magnitude of the difference between the 2 nutrients being comparable in the studies of Cerrate et al. (2019) (86% vs 59% k values for fat and protein ME) and Wu et al. (2019) (88% vs 57%). Finally, the equations of Wu et al. (2019) have been validated by the same authors and the same methodologies on additional NE measurements conducted on broilers on a small set of diets (Wu et al., 2019) and also in laying hens fed 16 diets (Barzegar et al., 2019b). Overall, the NE predictions proposed by Wu et al. (2019) would represent a first NE proposal for poultry feeds that would deserve additional validation in broilers, either obtaining new NE measurements data or running performance trials as performed in layers (Barzegar et al., 2020).

**Fig. 5:**
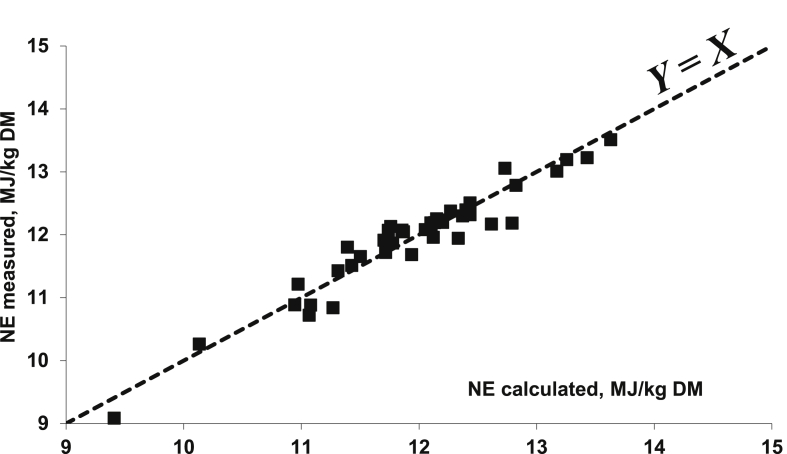
Relationship between measured NE of 41 pig diets at INRA facilities (de Lange et al., 2006a; Le Bellego et al., 2001; Le Goff et al., 2002; Noblet et al., 2001; van Milgen et al., 2001; unpublished data; mean = 11.95 MJ/kg DM; FHP = 750 kJ/kg BW^0.60^) and their NE values calculated according to Eqs. (Eq. 2), (Eq. 4)) (mean of both) of Noblet et al. (1994a); the correlation coefficient between both sets of values is 0.96. NE = net energy; FHP = fasting heat production.

**Fig. 6:**
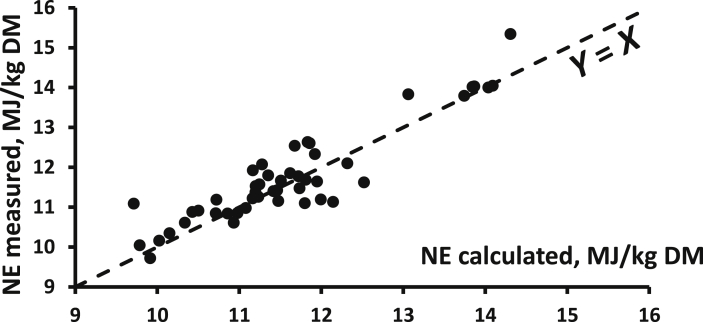
Relationship between measured NE of 46 diets at China Agricultural University in Beijing (mean: 11.75 MJ/kg DM; Li et al., 2018a) and NE as calculated from NE Eqs. (Eq. 2), (Eq. 4)) (mean of both) of Noblet et al. (1994a) (mean: 11.61 MJ/kg DM); the measured NE were adjusted for a common value of FHP equal to 750 kJ/kg BW^0.60^ as in the data of Noblet et al. (1994a). The correlation coefficient between both sets of values is 0.91. NE = net energy.

In pigs, the first NE system that has still some application today was proposed by Schiemann et al. (1972). It was based on indirect calorimetry measurements in heavy pigs (>100 kg) depositing predominantly fat. It has been the basis of the NE systems developed by CVB. Another NE system was proposed by Just (1982) in Denmark from measurements conducted on growing pigs using the CST method. This system has been replaced in Denmark by the so-called “Potential Physiological Energy " NE system based on in vitro digestion indicators and biochemical parameters. The last major NE system for pigs was proposed by Noblet et al. (1994a), from measurements on 61 diets fed to lean growing pigs. Its evaluation on a new set of NE measurements by the same authors and also measurements conducted by IVVO in The Netherlands is illustrated on Figs. 3 and 5. After 2010, an impressive series of NE measurements were done in China (Li et al., 2018b) on growing pigs with their results reported in Fig. 6. The 3 figures indicate a satisfactory correlation between measured NE values in growing pigs and NE values as calculated from the initial NE equations of Noblet et al. (1994a). In addition, for all 3 figures, the difference between measured and calculated NE values is not related to any chemical indicator of the feed, suggesting no systematic bias in the equation proposed by Noblet et al. (1994a). These equations were also confirmed in other NE studies (van Milgen et al., 2001) conducted in growing and heavier pigs (Noblet et al., 1994b) as well as in adult sows fed close to their maintenance requirements (Noblet et al., 1994b). This means that the NE prediction equations obtained for growing pigs can be applied to other stages of pig production (Noblet and van Milgen, 2004). Overall, the equations proposed by Noblet et al. (1994a) and other comparable equations such as the equation used in CVB Tables (2018) have become widely used in the pig feed industry and included in major feeding tables, for instance, NRC (2012), Rostagno et al. (2017) for Brazilian Feeding Tables and INRAE-CIRAD-AFZ (2019).

Another method for evaluating and validating an energy system involves the comparison of diets formulated on NE values with other energy values, such as DE or ME for the production performance outcomes. Unfortunately, little information has been published for poultry (Barzegar et al., 2020). On the other hand, data from several validation trials are available for comparing the interests of the different available energy systems for pigs. The results from 2 of them are reported in Table 6 that clearly indicate that the level of growth performance is better related to the diet NE intake than to their DE or ME intakes (Noblet and van Milgen, 2004). However, in the interpretation of such performance trials, attention should be paid to the precise characterization of performance; in the case of growing-finishing pigs, the most important point is the control or adjustment of performance for the same chemical or tissular composition of BW gain. From that point of view, the development of the so-called “Caloric efficiency” concept based on only growth and feed efficiency (Cemin et al., 2020) is suffering from important limitations which do not allow it to be used as a tool for NE evaluation (Zhang et al., 2020).

### 7.4 Evaluation of NE of ingredients

As for DE and ME, the NE value of ingredients can be measured directly, by the difference method, or by regression methods. For the difference method, the calculations described for DE/GE or ME/GE or ME/DE should be used for NE/ME with the following formula:

(see Eq. 1 to 4 for explanations) NE/ME (%) = 100 × NE_0_/ME_0_ = 100 × {NEtd – [NEbd/(1 - %MVbd)]} × (1 - %ti - %MVtd)/{{MEtd - [MEbd/(1 - %ti - %MVbd)]} × (1 - %MVtd)} .

The direct or difference approaches based on indirect calorimetry measurements have been implemented on an important series of ingredients fed to growing pigs (Li et al., 2017, 2018b) and also on a few major ingredients fed to poultry (Liu et al., 2017). The multiple regression method has been much less frequently used, either in poultry (Cerrate-Fernandez et al., 2012) or in pigs (Noblet et al., 1993a; van Milgen et al., 2001). The advantages and disadvantages of these techniques for NE measurement are the same as for DE or ME measurement. However, the regression method is better adapted to NE measurement than the direct or the difference method. Indeed, it has been stressed that, in order to be valid, NE measurements should be conducted on balanced diets fed at standardized feed intake and environmental conditions. These conditions are not easy to meet, especially for balancing the diets, in the direct and difference methods with either too low or excessive protein levels and/or too high fibre contents in the diets fed to the animal. In addition, the NE value of the raw material calculated may correspond to a predominant deposition of protein or fat, which differs from the average composition of BW gain in growing animals. Finally, the NE value obtained using the difference method cumulates the measurement errors and inaccuracies of DE, ME and HP (or RE) measurements. Overall, it is then suggested to use preferentially the (multiple linear) regression methods for evaluating NE values of ingredients using diets that are nutritionally balanced and normally consumed for optimizing the performance of animals.

The evaluation of NE content or any NE system is based on measurements conducted on complete diets. So, does the application of an NE equation obtained on diets to an ingredient produce a reliable NE value for this ingredient? A very limited number of publications answer that question. For pigs, Noblet et al. (1993a) measured the DE, ME and NE of 17 diets prepared from 13 ingredients and allowing the evaluation of DE, ME and NE contents of these ingredients by multiple linear regression. Their NE could also be calculated according to the different NE prediction equations proposed by Noblet et al. (1994a). Fortunately, the agreement between the 2 sets of NE values of the 13 ingredients was quite satisfactory, meaning that the NE equations obtained from measurements on diets were applicable to ingredients. These first results were confirmed later on a limited number of ingredients (Liu et al., 2017; van Milgen et al., 2001) and by the numerous studies compiled by Li et al. (2018b) for most potential ingredients used in pig feeds. There is not any comparable study for poultry. However, the study by Liu et al. (2017) on corn and soybean meal confirms that the equations published by Wu et al. (2019) give a close correlation between a predicted NE value and the measured NE value for these 2 contrasting ingredients.

While measurements of DE and, to a lesser extent ME, are relatively easy and can be undertaken on a large number of feeds at a reasonable cost, the actual measurement of NE requires specific expertise and is complex, expensive and time-consuming. Since the NE prediction equations (or NE systems) are applicable to both complete feeds and ingredients, the alternative to routine NE measurements is to use reliable NE prediction equations for calculating the NE value of any ingredient. Those based on DE or ME content and some chemical characteristics (Noblet et al., 1994a; Wu et al., 2019) (Table 5) can then be used directly from measured chemical composition and digestibility data provided by direct measurements, in vitro or near infra-red methods, or simply by using feeding tables. So, instead of tedious and rather useless efforts on routine NE measurements of ingredients, attention can be paid to important factors of variation of DE content in pigs and ME content in poultry for improving the NE prediction of ingredients.

**Table 6:** Performance of growing-finishing pigs according to energy system and diet characteristics.^1^^,^^2^

Item	DE	ME	NE
Added fat, % (Trial 1)			
0 (control)	100	100	100
2	100	100	100
4	99	99	100
6	98	98	100
Crude protein (30 to 100 kg; Trial 2)			
Normal (control)	100	100	100
Low	96	97	100
Crude protein (90 to 120 kg; Trial 3)			
Normal (control)	100	100	100
Low	97	98	100

1 Adapted from (Noblet and van Milgen, 2013).

2 Energy requirements [or energy cost of body weight (BW) gain] for similar daily BW gain and composition of BW gain; values are expressed relative to the energy requirement (or energy cost of BW gain) in the control treatment (considered as 100).

### 7.5 Expression of NE requirements of pigs and poultry

Energy requirements of pigs and poultry can either be expressed on the energy concentration of diets or, when animals are not fed ad libitum, on the daily supply of energy according to body weight, genotype, and stage of production. In the past, energy requirements for pigs were expressed on DE or ME basis and for poultry on ME basis. For a total of 61 diets fed to growing pigs, Noblet et al. (1994a) reported an average NE/ME ratio of 74% (and 71% for NE/DE), which also corresponds to the NE/ME ratio of a standard cereal-soybean meal diet. These authors proposed to move the DE and ME recommendations to NE recommendations by multiplying them by 0.71 and 0.74, respectively. Similarly, in broilers and based on a high number of diets, Carré et al. (2014) and Wu et al. (2019) obtained NE/AME and NE/AMEn ratios averaging 75% and 79%, respectively. As in pigs, the NE recommendations for poultry can then be obtained as AME or AMEn recommendations multiplied by 0.75 or 0.79, respectively. Finally, a slightly compromised method may involve calculating the NE/DE or NE/ME of each conventional diet adapted to each stage of production and breed and defining the NE recommendation for this diet for all other potential diets used at each stage.

As described above, the NE values of diets obtained in adult sows and the NE values of the same diets as calculated from the equations obtained in growing pigs are ranked similarly (Noblet et al., 1993b). A comparable conclusion is drawn for growing pigs weighing either 50 or 100 or 100 kg with no effect of BW on the ranking of diets (Noblet et al., 1994b). With a similar approach, Barzegar et al. (2019b) confirmed that broilers and laying hens use ME for NE quite similarly. These findings suggest that first, only one set of NE prediction equations, even though it was obtained initially in growing pigs at a specific stage of their growth, can be used in pigs at all stages of production and, second, the NE requirements can also be calculated as DE or ME requirements multiplied by 0.71 or 0.74 for all stages of production. In the case of poultry and, at least for broilers and laying hens, the rules for broilers can be applied to laying hens.

## 8 Conclusion

This review illustrates that the estimate of representative and reliable energy values of either complete feeds or ingredients for pigs and poultry requires accurate methods, although it may become more complex as feed energy evaluation moves from the DE or ME system to an NE system. However, even for DE or ME, there is a compelling need for standardization in the measurement protocol, the concepts and calculations to obtain comparable and reliable energy values applicable to modern production settings. For instance, it is suggested to standardize ME values to an N retention level close to what is observed in fast-growing animals fed continuously lowered dietary crude protein levels and associated higher proportions of dietary N that is retained in the body or exported in eggs or milk. The measurement of NE is rather complex and requires specific equipment and expertise. However, an NE system based on a validated prediction equation can be easily implemented from DE or ME values of feeds without the need for any further sophisticated measurement of NE values. This situation facilitates to a high extent the use of NE systems in both pig and poultry. Finally, it should be stressed that NE has a greater ability to differentiate feeds than DE or ME according to their true energy value and the actual response of the animals. As widely done in the pig sector, NE should be implemented in poultry with potential important and imminent improvements in accuracy of their nutrition and profitability of the sector.

## Author contributions

**Jean Noblet:** conceptualization, data curation, drafting manuscript; **Shu-Biao Wu:** critical review of the manuscript; **Mingan Choct:** critical review of the manuscript.

## Declaration of competing interest

We declare that we have no financial and personal relationships with other people or organizations that can inappropriately influence our work, and there is no professional or other personal interest of any nature or kind in any product, service and/or company that could be construed as influencing the content of this paper.
