# Corrigendum to “Methodologies for energy evaluation of pig and poultry feeds: A review” [Animal Nutrition volume 8 (2022), 185-203]

**DOI:** 10.1016/j.aninu.2022.05.002

**Published:** 2022-07-02

**Authors:** Jean Noblet, Shu-Biao Wu, Mingan Choct

**Affiliations:** a INRAE, UMR 1348 PEGASE, 35590 St-Gilles, France; b School of Environmental and Rural Science, University of New England, Armidale, NSW 2351, Australia

The authors have realized that an error repeated 4 times in this paper. The corrected version should be the following:

**At page 191, top left**

DCe (%) = 100 × DE_0_/GE_0_

=100 × {DEtd – [DEbd/(1 - % MVbd)] × (1 - %ti - %MVtd)}/{GEtd - [GEbd/(1 - %MVbd)] × (1 - %ti - %MVtd)}, (Eq. 2)

ME/DE (%) = 100 × ME_0_/DE_0_

=100 × {MEtd – [MEbd/(1 - % MVbd)] × (1 - %ti - %MVtd)}/{DEtd - [DEbd/(1 - %MVbd)] × (1 - %ti - %MVtd)}, (Eq. 3)

ME/GE (%) = 100 × ME_0_/GE_0_

=100 × {MEtd – [MEbd/(1 - % MVbd)] × (1 - %ti - %MVtd)}/{GEtd - [GEbd/(1 - %MVbd)] × (1 - %ti - %MVtd)}. (Eq. 4).

**At page 199, bottom right**

NE/ME (%) = 100 × NE_0_/ME_0_

=100 × {NEtd – [NEbd/(1 - % MVbd)] × (1 - %ti - %MVtd)}/{MEtd - [MEbd/(1 - %MVbd)] × (1 - %ti - %MVtd)}. (Eq. 6; see Eq. 1 to 4 for explanations).

We would like to apologise for this repeated mistake and we are really sorry for the inconvenience.

